# Anti-VGLUT2 autoantibodies associated with post-COVID neurocognitive dysfunction: a case report

**DOI:** 10.3389/fimmu.2025.1731744

**Published:** 2026-01-15

**Authors:** Jeyanthan Charles James, Bianca Teegen, Thivya Pakeerathan, Gregor Hütter, Theodoros Ladopoulos, Nadine Siems, Nadine Trampe, Ilya Ayzenberg, Ralf Gold, Simon Faissner

**Affiliations:** 1 Department of Neurology, St. Josef-Hospital, Ruhr-University Bochum, Bochum, Germany; 2 Clinical Immunological Laboratory Prof. Stöcker, Groß Grönau, Germany

**Keywords:** autoantibodies, autoimmune neurology, long COVID, neurocognitive dysfunction, post-COVID syndrome, vesicular glutamate transporter 2 (VGLUT2)

## Abstract

Post-COVID-19 syndrome (PCS) is frequently associated with fatigue and cognitive dysfunction, while underlying mechanisms remain unclear. We report a 44-year-old male with persistent symptoms following SARS-CoV-2 infection, including severe cognitive and motor fatigue, word-finding difficulties, and impaired concentration. Neuropsychological testing revealed marked deficits in alertness, attention, fluency, and processing speed. Serum analysis demonstrated anti-VGLUT2 autoantibodies. IVIG therapy yielded subjective but no objective improvement. This appears to be the first PCS case associated with VGLUT2 autoantibodies and raises the hypothesis of a potential pathophysiological link that requires confirmation in larger cohorts.

## Background

The condition, characterized by the persistence of symptoms or the emergence of new complaints at least three months after acute SARS-CoV-2 infection, lasting for at least two months without an alternative explanation, is subsumed under the terms post-COVID-19 condition or post-COVID syndrome (PCS) (1–3). Neurological manifestations are particularly common, with fatigue and cognitive dysfunction being the most frequently reported (4–6). Beyond these, neurological and neuropsychiatric PCS may include dysosmia, sleep disturbances, concentration deficits, memory impairment, sensory symptoms, pain, depression, and anxiety (7). The pathophysiological mechanisms underlying PCS and its neurological symptoms remain incompletely understood. Current hypotheses include direct viral invasion, systemic inflammation with complement activation, microglial activation, dysregulated coagulation, oxidative stress, autoimmune responses, and neurodegenerative processes (3, 8, 9).

Vesicular glutamate transporter 2 (VGLUT2) is a protein located in the synaptic vesicles of glutamatergic neurons and is responsible for transporting the neurotransmitter glutamate into these vesicles (10). Autoantibodies targeting this transporter, referred to as anti-VGLUT2 antibodies, have only recently been described. Emerging evidence suggests that such autoantibodies can be detected in patients with autoimmune neurological disorders. In a recently published larger cohort, VGLUT2 autoimmunity was associated with a spectrum of central and peripheral nervous system phenotypes, most commonly encephalitis, cognitive impairment, and sensorimotor neuropathies, while immunotherapy led to at best modest clinical improvement in most cases (11, 12). These findings support the concept of an autoimmune VGLUT2-associated neurological spectrum but also underline the heterogeneity of clinical presentations and treatment responses.

In the following, we report on a patient with PCS presenting with long-term neurocognitive impairment and serological evidence of VGLUT2 autoantibodies.

## Detection of anti-VGLUT2 autoantibodies

Serum and cerebrospinal fluid (CSF) samples were analyzed in a specialized clinical neuroimmunology laboratory. Testing for anti-VGLUT2 autoantibodies was performed using an indirect immunofluorescence assay (IIFA) on cryosections of monkey cerebellum as previously described (11). Briefly, patient serum was incubated on cerebellar sections at a starting dilution of 1:10 in phosphate-buffered saline containing 0.2% Tween-20 for 30 minutes at room temperature, followed by a washing step and incubation with a fluorescein-labeled (FITC) anti-human IgG secondary antibody. Fluorescence patterns were evaluated independently by two experienced observers.

**Figure 1:**
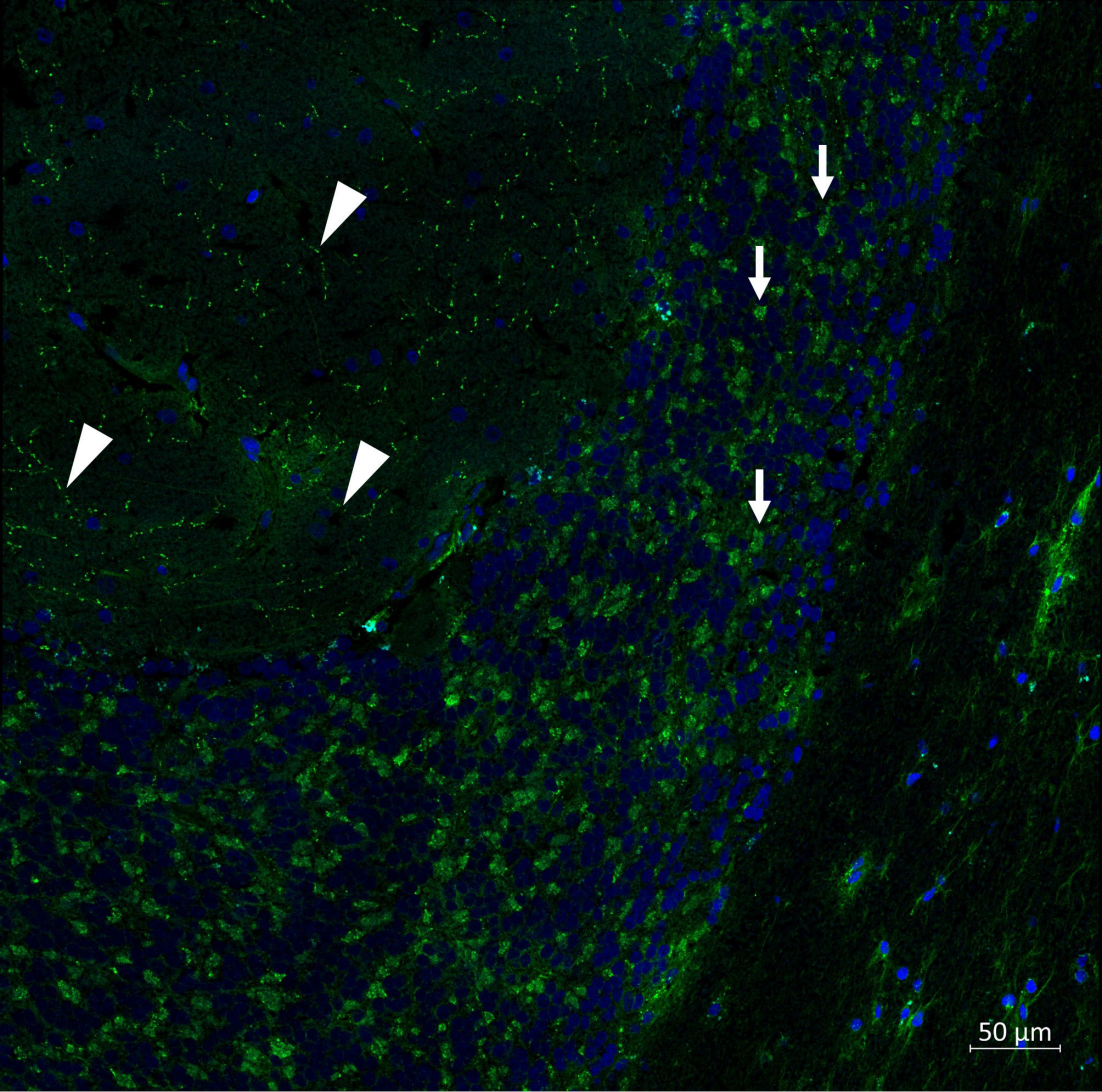
Immunofluorescence labeling of monkey cerebellum with patient serum (VGLUT2). Shown is an immunofluorescence staining of a monkey cerebellar section after incubation with patient serum at a dilution of 1:100 in PBS-Tween. Bound human IgG antibodies were detected using an Alexa Fluor 488–labeled anti-human IgG secondary antibody (green). Cell nuclei were counterstained with DAPI (blue). Arrows indicate the irregular, patchy fluorescence within the granular layer, whereas arrowheads highlight the discontinuous, streak-like fluorescent structures in the molecular layer. This staining pattern is consistent with the synaptic VGLUT2 signal described in previous reports. Scale bar: 50 µm.

For confirmation of specificity, a monospecific anti-VGLUT2 assay based on human VGLUT2-expressing cells was used as previously reported (11). Patient serum was incubated on fixed VGLUT2-transfected cells and on untransfected control cells, followed by incubation with the same secondary antibody as described above. Specific binding was defined by a clear fluorescence signal on VGLUT2-expressing cells in the absence of staining of untransfected cells. While this assay served to confirm antigen specificity, representative fluorescence images were not available for publication (cf. **Figure 1**).

## Case presentation

We report a 44-year-old male patient with no relevant pre-existing medical conditions or long-term medication who presented for the first time to our post-COVID outpatient clinic because of persistent symptoms. He had experienced a SARS-CoV-2 infection in July 2022, 22 months before the first presentation to our department. Since the acute infection, he had suffered from a broad spectrum of symptoms that markedly impaired his daily functioning. Clinically, the patient exhibited reduced physical and cognitive endurance, generalized pain, concentration difficulties, word-finding problems, and pronounced fatigue. He additionally reported dizziness during cognitive exertion, an increased need for breaks in his daily work routine, and occasional disorientation. Overall, these symptoms remained clearly limiting in everyday life and persisted up to the time of presentation.

Comprehensive neuropsychological assessment was conducted. Among other tests, the Test Battery for Attention (TAP) was used to assess alertness, and the Fatigue Scale for Motor and Cognition (FSMC) to quantify fatigue. Deep cognitive assessment was conducted using standardized procedures covering attention, processing speed, verbal fluency, short-term and working memory, and verbal as well as visual learning and memory functions. Across several domains, the patient achieved percentile ranks well below the normative range. Particularly striking were impairments in tonic alertness (markedly reduced), phonematic fluency, focused and selective attention, and motor processing speed; a typical trait in neurological PCS (6). The FSMC confirmed severe motor and cognitive fatigue.

Neurological examination revealed no focal deficits. Cranial MRI demonstrated isolated, nonspecific gliosis within the supratentorial white matter, otherwise age-appropriate. The cervical spinal cord appeared unremarkable. EEG showed a normal alpha rhythm without pathological potentials. Extended laboratory work-up revealed biochemical evidence suggestive of early vitamin B12 deficiency. Serum vitamin B12 was 356.0 pg/mL (reference range 197–771 pg/mL), i.e. in the low–normal range, while holotranscobalamin was slightly below the lower reference limit at 58.9 pmol/L (reference >60 pmol/L). Methylmalonic acid was within the normal range at 222.13 nmol/L (reference range 73–271 nmol/L), indicating no manifest cellular vitamin B12 deficiency at the time of testing. This constellation is compatible with early-stage or subclinical vitamin B12 deficiency without biochemical evidence of overt cellular deficiency. Vitamin B12 substitution was recommended.

Cerebrospinal fluid analysis showed normal cell count, a mildly elevated total protein of 49 mg/dl, and an albumin quotient of 8.1. No oligoclonal bands were detected. Extended serological testing for onconeural antibodies, antineural antibodies, aquaporin-4, and MOG antibodies was negative. Notably, VGLUT2 autoantibodies were detected in serum at a titer of 1:100 but were absent in cerebrospinal fluid. Key blood and CSF findings, including vitamin B12 status and anti-VGLUT2 titers at baseline and after 14 months of IVIG therapy, are summarized in **Table 1**.

**Table 1:** Summary of relevant laboratory and cerebrospinal fluid findings.

Parameter	Patient value	Reference range	Interpretation
Hemoglobin	14.1 g/dl	14.0-18.0	normal
MCV	92.2 fl	85.0-95.0	normal
MCH	30.7 pg	27.0-33.0	normal
Serum vitamin B12	356.00 pg/ml	197-771	low–normal
Holotranscobalamin	58.9 pmol/l	>60	slightly decreased
Methylmalonic acid	222.13 nmol/l	73-271	normal
CRP	<5mg/l	<5	normal
CSF cell count	5 cells/µL	<5	normal
CSF protein	49 mg/dl	15.0 – 45.0	slightly increased
Albumin quotient	8.1	age-adjusted <8.0	slightly increased
Anti-VGLUT2 serum titer (baseline)	1:100	negative (no established quantitative reference range)	positive
Anti-VGLUT2 serum titer (14 months)	1:320	negative (no established quantitative reference range)	positive, persistent
Anti-VGLUT2 CSF titer	negative	negative (no established quantitative reference range)	normal

Therapeutically, the patient initially received antihistamines, which were associated with a transient subjective improvement of symptoms. Following the detection of VGLUT2 autoantibodies, intravenous immunoglobulin (IVIG) therapy (1 g/kg body weight) was initiated and administered at regular intervals over a period of 14 months. During IVIG treatment, the patient reported further subjective amelioration of symptoms. However, neuropsychological reassessment after 14 months revealed a largely unchanged cognitive performance profile without objective improvement. Serologically, VGLUT2 autoantibodies remained persistently detectable at high titers (1:320).

## Conclusion/Outlook

A growing body of evidence suggests that a spectrum of central and peripheral nervous system disorders may be associated with the presence of VGLUT2 autoantibodies. Screening of 314 patient sera with a distinct uncharacterized neuronal IIFA pattern between 2016 and 2024 identified vesicular glutamate transporter 2 (VGLUT2) as the novel target, confirmed in 285 cases by multiple independent assays. Clinical data showed that VGLUT2 autoimmunity most often presented with encephalitis, cognitive impairment, or polyneuropathy, with index patients displaying predominant cognitive deficits, sensorimotor disturbances, gait abnormalities, and frequent comorbid type 2 diabetes. Although immunotherapy led to only modest improvement in most cases, these findings suggest that anti-VGLUT2 autoantibody-associated syndromes represent a distinct form of autoimmune neurological disease (11).

In this context, we report for the first time a patient with neurological post-COVID syndrome, presenting with severe mixed fatigue and marked neuropsychological deficits affecting components of attention, like concentration, and working memory, in whom the detection of VGLUT2 autoantibodies may provide a plausible pathophysiological correlate. At present, we have not yet conducted a systematic investigation of larger cohorts of PCS patients or healthy control subjects for anti-VGLUT2 autoantibodies at our center, so there are no comparative data between anti-VGLUT2-positive and -negative PCS patients or healthy individuals. This case report therefore cannot provide prevalence estimates or direct comparisons and must be considered hypothesis-generating.

Several limitations need to be acknowledged. First, this is a single case report and can therefore only generate hypotheses regarding a possible association between VGLUT2 autoimmunity and PCS-related neurocognitive dysfunction. Anti-VGLUT2 antibodies were detected only in serum and not in cerebrospinal fluid, which limits conclusions about a directly pathogenic intrathecal immune process. Furthermore, we did not include parallel immunofluorescence images from healthy control subjects or anti-VGLUT2-negative PCS patients, which represents a methodological limitation. Finally, despite 14 months of IVIG therapy, repeated neuropsychological assessment did not show objective cognitive improvement, so no firm conclusions can be drawn about either the pathogenic relevance of the antibody or the efficacy of IVIG in this context.

Taken together, our observation supports the notion that anti-VGLUT2 autoimmunity may occur in the setting of neurological PCS and might represent one of several overlapping mechanisms contributing to persistent neurocognitive symptoms. However, a single case is far from sufficient to justify routine screening for anti-VGLUT2 antibodies in all PCS patients. Systematic studies in larger cohorts are needed to determine the prevalence, clinical relevance, and therapeutic implications of VGLUT2 autoimmunity in PCS and to identify those patients in whom targeted antibody testing may be most informative.

## Data Availability

The original contributions presented in the study are included in the article/supplementary material. Further inquiries can be directed to the corresponding author.
